# A unified global costing framework catalyzes strategic investment in rice breeding

**DOI:** 10.3389/fpls.2025.1681605

**Published:** 2026-01-16

**Authors:** Sanjay K. Katiyar, Lekha T. Pazhamala, Reshmi Rani Das, Lennin Musundire, Atugonza Bilaro, Theodore T. Kessy, Maxwell Darko Asante, Ram Baran Yadaw, Girish Chandel, Abhinav Sao, Amrit Prasad Poudel, Kirpal Agyemang Ofosu

**Affiliations:** 1 Genetic Diversity and Improvement, Africa Rice Center (AfricaRice), Bouake, Côte d’Ivoire; 2 Rice Breeding Innovations, International Rice Research Institute (IRRI), Manila, Philippines; 3 Accelerated Breeding Initiative, Genetic Innovation, Consultative Group on International Agricultural Research, CIMMYT, Hyderabad, India; 4 International Maize and Wheat Improvement Center (CIMMYT), Nairobi, Kenya; 5 Accelerated Breeding Initiative, Genetic Innovation, Consultative Group on International Agricultural Research, CIMMYT, Nairobi, Kenya; 6 Tanzania Agricultural Research Institute, TARI-IFAKARA (KATRIN), Morogoro, Tanzania; 7 CSIR-Crops Research Institute (CSIR-CRI), Kumasi, Ghana; 8 National Rice Research Program (NRRP), Nepal Agricultural Research Council, Hardinath, Nepal; 9 Indira Gandhi Krishi Vishwavidyalaya, Raipur, Chhattisgarh, India

**Keywords:** rice breeding, cost analysis, genetic gain, breeding modernization, economic benchmarking, strategic investments, food security

## Abstract

Accelerating research investment and breeding innovation is critical to strengthening food system resilience and tackling the escalating food crisis across the Global South. To deliver transformative impact, rice breeding programs must transition into modern, focused, and data-driven systems that drive both financial and operational efficiency. Until recently, systematic cost assessment of rice breeding pipelines, crucial for strategic resource allocation, faster genetic gains, and enhanced varietal development, has been largely overlooked. To bridge this gap, we developed the Unified Global Costing Framework for Rice Breeding (UGCF-Rice), a standardized system for data collection, cross-program benchmarking, identifying key cost drivers, efficiency gaps, and opportunities for optimization. The framework’s effectiveness was demonstrated through case studies costing four National Agricultural Research and Extension System (NARES) rice breeding pipelines in South Asia (India and Nepal) and Sub-Saharan Africa (Tanzania and Ghana), using UGCF-Rice in conjunction with the University of Queensland’s Breeding Program Costing Tool (UQ-BPCT). This comprehensive analysis revealed that rice breeding pipeline costs ranged from USD 26,781 to 39,221, excluding institutional overheads and cross-cutting charges. Costing of two restructured pipelines integrating speed breeding technologies, demonstrated that strategic modernization can greatly enhance efficiency, achieving a 2.3-fold reduction in breeding cycle time, a 17–24-fold increase in throughput (fixed lines per cross), and a 1.6–20-fold reduction in land use compared to conventional breeding. This global costing framework establishes a data-driven foundation for strategic research investment and optimization, empowering policymakers, donors, and breeders to enhance efficiency, sustainability, and impact in rice breeding. Building on these insights, we propose an integrated “Cost-efficient Rice Breeding and Innovation Model” to empower NARES for global impact.

## 1 Introduction

The global rice food system stands at a critical crossroads, facing escalating food and nutrition insecurity. Meeting future demand will require an additional 75 million tons of rice by 2050, even as per capita land availability is projected to decline by 25% (Kruseman et al., 2020; CGIAR, 2024; Pede et al., 2024). Current genetic gains remain insufficient to meet this growing demand, further constrained by diminishing returns on R&D investments. Positioning rice at the core of sustainable agriculture, CGIAR is driving a global transformation toward climate-smart innovation, harnessing technology to accelerate varietal development (CGIAR, 2024).

Rice breeding innovations, such as speed breeding, smart breeding, digitization, genotyping, precision phenotyping, and AI-powered genomic selection, enable shorter breeding cycles and faster delivery of climate-resilient, nutrient-rich cultivars (Atlin et al., 2017; Katiyar et al., 2025; O’Connor et al., 2013; Heffner et al., 2010; Ahmed et al., 2022; Alemu et al., 2024; Seck et al., 2024). Breeding Modernization (BM) represents a systemwide transformation anchored in breeding and operational excellence, is essential for transforming rice breeding programs globally. It replaces traditional, labour-intensive practices with data-driven, technology-enabled strategies that optimize the breeder’s equation by enhancing selection intensity, accuracy, and genetic variance while accelerating cycle time (Nguyen et al., 2023; Juma et al., 2021; Atlin et al., 2017; Cobb et al., 2019; Katiyar et al., 2023; Katiyar et al., 2025; CGIAR Excellence in Breeding, 2023; CGIAR Initiative on Accelerated Breeding, 2023; CGIAR Excellence in Breeding, 2022; International Rice Research Institute, 2022). In recent years, IRRI’s global rice breeding modernization efforts have demonstrated measurable genetic gains (Cobb et al., 2019; Bhosale et al., 2025; Brzozowski et al., 2022; Seck et al., 2024). In contrast, many public-sector NARES breeding programs in Sub-Saharan Africa and South Asia still rely on conventional methods, constrained by limited funding, outdated infrastructure, and fragmented systems. This highlights the urgent need for strategic investment and efficient resource use to modernize NARES breeding pipelines to unlock their full potential. Under global modernization efforts such as the CGIAR Accelerated Breeding Initiative (ABI), systematic costing has emerged as a key driver of efficiency, sustainability, and impact across different crop breeding networks. It enables identification of major cost drivers, estimation of return on investment (ROI), and strategic donor engagement. However, despite its growing importance, the absence of a standardized framework limits consistent data collection, cross-program benchmarking, and identification of efficiency gaps and optimization opportunities.

Structured costing of rice breeding pipelines is fundamental for optimizing resources, guiding strategic investments, and improving operational efficiency in modern, data-driven breeding systems. To address this need, we developed a Unified Global Costing Framework for Rice Breeding (UGCF-Rice), a comprehensive model that captures both direct and indirect costs across all stages of the breeding pipeline, including personnel, field and laboratory operations, genotyping, phenotyping, equipment, infrastructure, and key inputs. Integrated with the UQ-BPCT, the UGCF-Rice enables systematic cost analysis to identify major cost drivers, uncover inefficiencies, and highlight opportunities to improve breeding efficiency, scalability, and impact. The framework was applied to assess the cost structures of four NARES rice breeding pipelines: two in Sub-Saharan Africa (Tanzania and Ghana) and two in South Asia (India and Nepal) and to benchmark efficiencies across conventional and modernized breeding systems. This system-level analysis establishes a strong evidence base to drive cost-efficient innovation, optimize breeding operations, and guide high-impact, performance-driven investments and strategic partnerships.

## 2 Methods

### 2.1 Establishing a unified global costing framework to drive efficiency in rice breeding

To enable comprehensive and comparative costing of rice breeding pipelines across countries and regions, we developed the Unified Global Costing Framework (UGCF-Rice) - the first standardized and scalable model for mapping and costing rice breeding programs globally. This framework provides a reproducible and transparent basis for cross-program cost analysis and benchmarking across diverse breeding systems and operational contexts. The framework provides a robust platform for detailed financial analysis and benchmarking, employing a structured, stepwise methodology that begins with defining the breeding program context and systematically mapping the entire breeding scheme, from germplasm development through testing and evaluation to varietal release.

**Figure 1:**
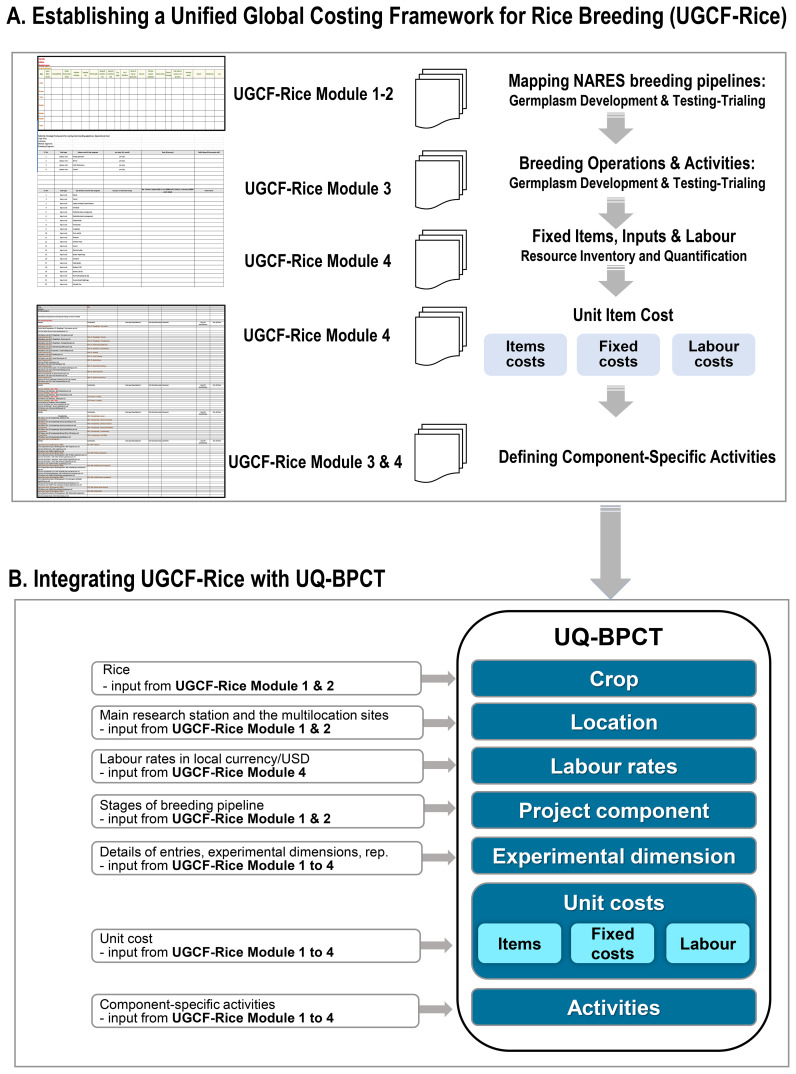
Development of ‘Unified Global Costing Framework for Rice Breeding (UGCF-Rice)’. **(A)** The figure illustrates a structured process for capturing and organizing data using UGCF-Rice to support accurate costing. It uses standardized modules to define pipelines, list operations and inputs, and assign unit costs. **(B)** These inputs feed into the University of Queensland’s Breeding Program Costing (UQ-BPCT) tool, integrating key elements: crop, location, labor rates, project components, experimental design, and activities, for a comprehensive cost analysis.

Each pipeline was meticulously deconstructed into discrete operational components, covering all major and minor breeding activities starting from parental selection, crossing, early and advanced generation selection, multi- environment trials up to product advancement. For each activity, the framework mandates detailed documentation of the required inputs, input quantities and unit costs. These were recorded using standardized UGCF-Rice Module 1 to 4 (**Supplementary Tables S1-S4**), ensuring consistency and comparability across breeding programs. An illustrative overview of this Unified Global Costing Framework for Rice Breeding (UGCF-Rice) is provided in **Figure 1**.

### 2.2 Systematic data capture using UGCF-Rice

The UGCF-Rice is composed of user-friendly, customizable Excel-based templates that facilitate detailed data capture across all stages of the breeding pipeline. UGCF-Rice Module 1–2 records the detailed processes involved in germplasm development and the testing and trial phases. UGCF-Rice Module 3 itemizes breeding operations and activities by stage, and UGCF-Rice Module 4 catalogues all required inputs, their unit costs, and quantities (**Figure 1A**). UGCF-Rice Module 1 to 4 (**Supplementary Tables S1-S4**) collectively form an all-inclusive data capture system that enables rice breeding programs to systematically document stage-specific operational and financial components across the breeding pipeline. These inputs are then integrated into the University of Queensland Breeding Program Costing Tool (UQ-BPCT; https://aussorgm.org.au/downloads/breeding-costing-tool/), where each activity and input are aligned with corresponding costing modules (**Figure 1B**).

Data represent actual expenditures incurred by each National Agricultural Research and Extension System (NARES) during the main crop season of the 2024 fiscal year. Costs were originally recorded in local currencies and converted to USD using exchange rates as of 27 May 2025 (1 USD = INR 85.3; 1 USD = NPR136.53; 1 USD = TZS 2698.5; 1 USD = GHS 10.43). The study was designed to capture costs within short and overlapping periods across study sites in the same year, rather than over extended periods, to minimize inflationary effects and ensure a consistent baseline for cross-program benchmarking on a globally comparable scale. The UGCF-Rice framework provides a robust foundation for harmonized cost capture, strategic investment decisions, and continuous optimization. However, the estimates did not account for institutional overheads and cross-functional cost variations, potentially resulting in a modest underestimation of the total program costs.

## 3 Results

### 3.1 A unified global costing framework for rice breeding

A Unified Global Costing Framework (UGCF-Rice) was developed to standardize and strengthen the financial assessment of rice breeding pipelines globally. This modular framework comprises four key components designed to capture precise data across all stages of the breeding process, from germplasm development through testing, trialing, and varietal release. Data are systematically recorded using intuitive Excel-based templates, described in detail in the following sections.

#### 3.1.1 UGCF-rice module 1: germplasm development phase

UGCF-Rice Module 1 was specifically designed and developed to capture all critical parameters related to germplasm development, spanning the entire early-stage breeding process, from parental selection through to the fixation of lines in F_5_/F_6_/F_7_ (**Supplementary Table S1**). Each of these stages is a component of the pipeline and is costed separately as an individual project, referred to as a ‘project component’ in the costing tool. This exhaustive template ensures that key operational details, including season, planting method, population size, spatial layout, selection criteria, and advancement rates at each stage of the breeding program are systematically documented. The parameters recorded include the number of entries, population size, plot dimensions, plant-to-plant and row-to-row spacing, row length, number of plants per row, number of rows per entry/population, total rows, total land area required, selection percentage, number of lines advanced to the next generation, and harvest method. This level of granularity enables accurate estimation of ‘experimental dimensions’ that support the calculation of resource requirements and facilitate standardization across breeding programs.

#### 3.1.2 UGCF-rice module 2: testing and trialing phase

UGCF-Rice Module 2 is structured to capture the critical parameters associated with the testing and trialing phases. It records trial stages, the number of test entries and checks, the experimental design, replications, the number and location of trial sites, plot dimensions, and the total number of plots. It also includes statistical and analytical metrics such as the standard error of difference (SED), coefficient of variation (CV), critical difference (CD), analysis of variance (ANOVA), and heritability estimates. Furthermore, this template accommodates phenotypic and genotypic screening data for evaluation under specific traits or stress conditions, ensuring that trial performance and genetic gain assessments are effectively integrated into the standardized costing framework (**Supplementary Table S2**).

#### 3.1.3 UGCF-rice module 3: breeding operations and activities across the pipeline stages

UGCF-Rice Module 3 captures the full spectrum of breeding operations at each stage, from parental selection to elite line advancement, by detailing both primary activities and their corresponding sub activities (**Supplementary Table S3**). It differentiates shared operations across germplasm development and testing and trialing (e.g. land preparation, seed preparation, nursery raising, and transplanting) and breaks these down into granular tasks such as field cleaning, ploughing (preseason, nursery bed, or transplanting), rotavating, and land levelling. For each operation, the template records the number of labourers required to perform an activity (e.g. crossing or hybridization, preharvest observation, postharvest observation or seed processing), frequency (e.g. number of times a particular activity/sub activity is performed such as irrigation, manual weeding, etc.), duration (hours per day), and scale (per unit area), thus allowing breeders to precisely quantify operational demands and benchmark them across locations or seasons.

#### 3.1.4 UGCF-rice module 4: capital investments and input costs across the breeding pipelines

UGCF-Rice Module 4 consolidates the complete operational costs, including item costs, labour costs and fixed costs, providing a detailed inventory of consumables, stationary, supplies, equipment, labour, and service costs (**Supplementary Table S4**). The costs of all items such as fuel, electricity, seed packets, field labels, agrochemicals (fertilizers, herbicides, pesticides, etc.) that are consumed during the crop season, as well as all field supplies, and one-time costs such as seed shipping courier charges to multiple sites for testing, service costs for genotyping or quality trait assessment, are categorized as input costs. Capital/Fixed costs, on the other hand, refer to items that can be used for more than one season across several components and product profiles, such as vehicles, equipment, tools, machines, and infrastructure (e.g. cold rooms, crossing chambers, and storage rooms). Fixed costs also include the salaries of permanent staff such as breeders or scientists, which are difficult to assign to a particular component and are therefore shared across components and product profiles. This comprehensive cost structure ensures that breeding programs can accurately estimate and compare cost drivers, assess cost efficiency, and identify opportunities for optimization.

### 3.2 Comparative mapping of NARES rice breeding pipelines in South Asia and Sub-Saharan Africa

We mapped the broad structures of four representative lowland irrigated rice breeding pipelines from leading NARES programs, including the TARI (Tanzania Agricultural Research Institute, Tanzania) and CRI (CSIR-Crops Research Institute, Ghana) in Sub-Saharan Africa and IGKV (Indira Gandhi Krishi Vishwavidyalaya, India) and the NRRP (National Rice Research Program, Nepal) in South Asia. These pipelines were strategically selected from an initial pool of twelve documented pipelines to reflect the structural and operational diversity of regional breeding programs. This analysis establishes a strong foundation for cross-program benchmarking, strategic resource allocation, and informed investment planning.

**Figure 2:**
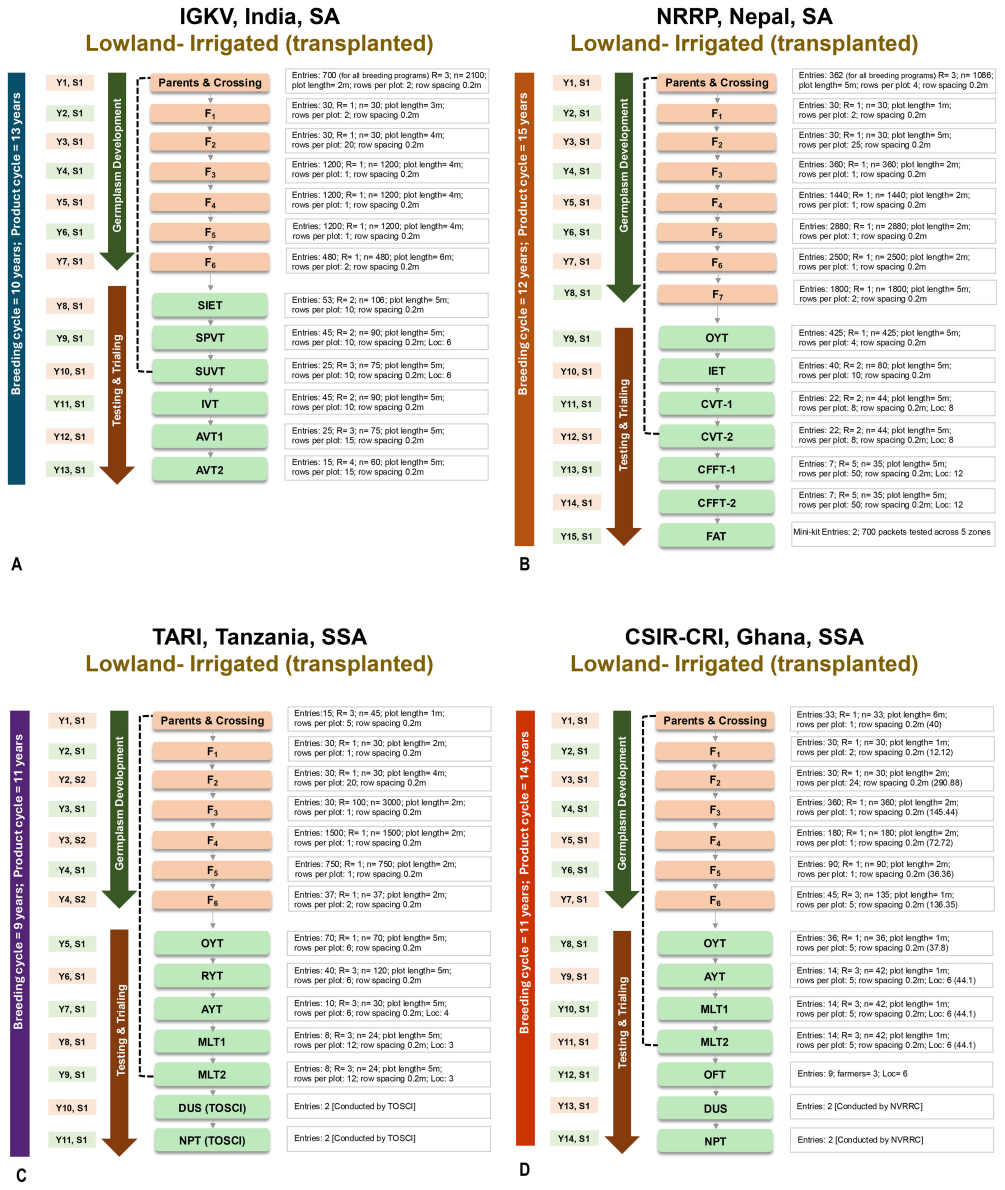
Comparative mapping of NARES rice breeding pipelines in South Asia and Sub-Saharan Africa. The figure maps lowland irrigated rice breeding pipelines at **(A)** IGKV (India), **(B)** NRRP (Nepal), **(C)** TARI (Tanzania), and **(D)** CSIR-CRI (Ghana), highlighting key stages, timelines, and activities. It outlines germplasm development, testing phases, breeding and product cycles, and evaluation sequences. Institutional variations in structure and trialing approaches offer a cross-regional view of NARES breeding practices.

Most NARES rice breeding programs in South Asia and Sub-Saharan Africa still depend on conventional pedigree methods, which are characterized by long breeding cycles, low-cost efficiency, and high labour intensity. The IGKV pipeline comprises 13 components, spanning a 10-year breeding and 13-year product cycle (**Figure 2A**; **Supplementary Tables S5, S6**). The NRRP, which is the most complex, includes 15 components with line fixation up to F_7_, requires 12 years for breeding and 15 years for product delivery (**Figure 2B**; **Supplementary Tables S7, S8**). The TARI and CRI pipelines each involve 14 components (**Figures 2C, D**; **Supplementary Tables S9–S12**) but differ in duration; the TARI completes breeding cycle in 9 years and product development in 11, while the CRI takes 11 and 14 years, respectively. None of these pipelines are modernized or strategically optimized to increase cost efficiency or accelerate genetic gains.

In India, the All India Coordinated Rice Improvement Project (AICRIP) has implemented a robust, tiered evaluation framework beginning with Initial Varietal Trials (IVT), advancing through Advanced Varietal Trials (AVT-1 and AVT-2), and encompassing extensive multilocation, multiyear assessments for yield potential, stress resilience, and grain quality. Nepal’s NRRP, under the Nepal Agricultural Research Council (NARC), follows a comparable stage-gate evaluation protocol, comprising Coordinated Varietal Trials (CVT1 and CVT2), Coordinated Farmers’ Field Trials (CFFT1 and CFFT2), and Final Advanced Trials (FATs), with strong emphasis on participatory validation under real-world farming conditions to enhance relevance and adoption. In Sub-Saharan Africa, the Tanzania Agricultural Research Institute (TARI) conducts a rigorous multitiered evaluation process, initiating with Advanced Yield Trials (AYT) and progressing through multilocation trials (MLT-1 and MLT-2) to evaluate yield performance, stress tolerance, and grain quality. The final stage of the National Performance Trials (NPT) is independently conducted by the Tanzania Official Seed Certification Institute (TOSCI), which serves as the critical gateway for varietal release. Similarly, Ghana’s National Variety Release and Registration Committee (NVRRC) oversees the evaluation, release, and registration of new varieties, ensuring delivery of regionally adapted, high-performing cultivars. These evaluation systems integrate scientific rigor, regulatory oversight, and stakeholder participation to ensure that released varieties are high yielding, resilient, and well suited to local agro-ecologies.

### 3.3 Cost benchmarking of NARES rice breeding pipelines in South Asia and Sub-Saharan Africa

**Figure 3:**
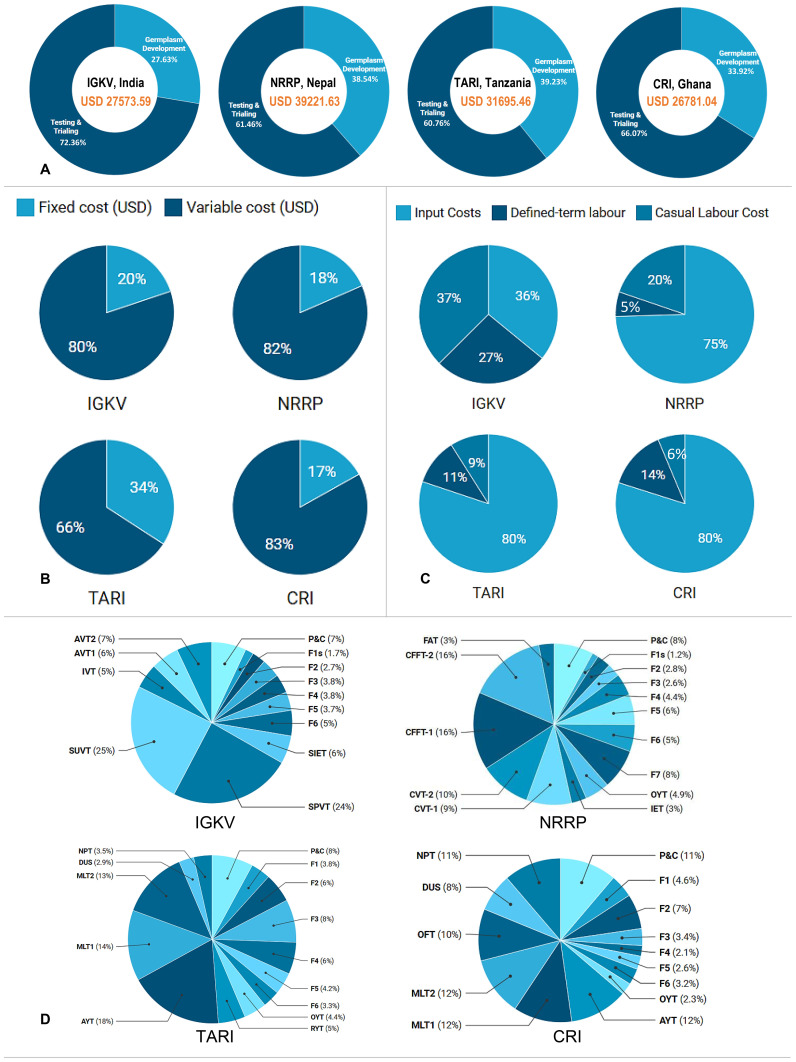
Cost benchmarking of NARES rice breeding pipelines in South Asia and Sub-Saharan Africa. **(A)** Total breeding pipeline costs (USD) and cost distribution between germplasm development and testing phases for IGKV, NRRP, TARI, and CSIR-CRI. **(B)** Comparison of the proportion of fixed and variable costs across NARES pipelines. **(C)** Breakdown of variable costs into inputs, defined-term labor, and casual labor, highlighting institutional differences in cost structure. **(D)** Percentage allocation of total costs across key breeding stages and activities.

A comparative cost analysis across four national rice breeding programs revealed substantial variability in total pipeline expenditures, ranging from USD 26,781 in Ghana to USD 39,221.60 in Nepal, with an average of USD 31,317.90. Nepal’s breeding program incurred the highest pipeline investment, followed by Tanzania (USD 31,695.50), India (USD 27,573.60), and Ghana (**Figure 3A**). These cost differentials underscore the influence of divergent breeding strategies, institutional capacities, programmatic scales, and underlying cost architectures. The cost estimates reflect only the direct and operational expenditures incurred during the execution of breeding pipeline activities, excluding institutional overheads, administrative charges, technological investments, and other cross-cutting expenses that would significantly increase total costs. To ensure precision and practical relevance for breeders, the input requirements were standardized for a 4,000 square-metre (sq. m) area (equivalent to one acre) and subsequently scaled to per- sq. m costs. Fuel expenses related to outstation travel for monitoring multilocation trials, along with land lease costs, were equitably apportioned across all relevant trialing stages, such as AYT, MLT1, and MLT2, to enable accurate cost allocation throughout the pipeline.

Strategic cost analysis across four representative rice breeding pipelines in South Asia and Sub-Saharan Africa reveals that germplasm development, including crossing, early-generation advancement, and line fixation, accounts for a substantial 28% to 39% of total pipeline expenditures, underscoring its role as a critical resource-intensive phase within the breeding continuum. In contrast, the testing and trialing phases, including Stage 1 trials, preliminary yield trials, trials in multiple locations, on-farm evaluations, Distinctiveness, Uniformity, and Stability (DUS) assessments, and national performance trials, dominated overall costs, consuming 61% to 72% of total resources (**Figure 3A**). This high investment reflects the operational complexity, logistical intensity, and stringent monitoring required to generate high-quality, decision-grade data across spatially diverse and ecologically variable target populations of environments (TPEs).

#### 3.3.1 Optimizing fixed and variable cost dynamics in rice breeding

To elucidate the underlying cost drivers, we conducted a detailed breakdown of total expenditures into fixed and variable components; the latter include three principal categories, namely, input, defined-term labour, and casual labour cost. The analysis also considered the effects of the breeding program scale, infrastructure capacity, and access to institutional support services on overall expenditure patterns under fixed costs.

Fixed costs constituted 17-34% of total expenditures (avg. 22%), whereas variable costs dominated at 66-83% (avg. 78%), reflecting the operationally intensive nature of NARES rice breeding programs (**Figure 3B**). Among the variable costs, field input expenses, such as labels, bags, and charges for multilocation trials (NPT, DUS, OFT, seed shipment, and outsourced quality assessments), constituted the largest component, averaging 67.8%, especially in Sub-Saharan Africa. Labour costs followed, typically accounting for 20-64% of the total cost. In the NRRP (Nepal), TARI (Tanzania), and CRI (Ghana), input costs make up 75–80% of variable expenses. In contrast, India showed a labour-dominated structure, with labour comprising 64% and inputs comprising 36% of the variable costs (**Figure 3C**). These trends highlight the need for region-specific cost optimization, aligned with local labour dynamics, input costs, and mechanization levels, to sustain genetic gains and breeding efficiency.

#### 3.3.2 Stage-specific cost profiling of rice breeding pipelines

Stagewise dissection of total breeding pipeline expenditures revealed consistent patterns across institutions in South Asia (SA) and Sub-Saharan Africa (SSA), shaped predominantly by regional variations in pipeline design, operational intensity, and breeding methodologies. The pipeline was structurally segmented into four key functional stages: (i) parental selection and crossing (P&C), (ii) early-generation advancement and germplasm development (F_1_-F_6_), (iii) on-station yield evaluations from preliminary to advanced stages, and (iv) multi environment testing (MET).

Parental selection and crossing (P&C) constituted a strategic entry point in the breeding continuum, accounting for 7-11% of total pipeline expenditures, with an average of 8.5% (**Figure 3D**). This stage integrated hybridization activities, fixed infrastructure, and labour costs. In India and Nepal, traditional crossing blocks were extensive, comprising 300–700 parental lines, including landraces, gene bank accessions, and trait-specific donors, serving multiple breeding pipelines simultaneously. In contrast, breeding programs in Tanzania and Ghana operated more streamlined crossing systems, deploying tailored parent sets aligned with the specific objectives of each pipeline. Early generation advancement (line fixation) represented a major cost-intensive phase, absorbing 22-31% of the total pipeline costs, averaging approximately 27.05%. This stage encompassed advancement from the F_1_ to the F_6_/F_7_ generations via pedigree or bulk selection strategies defined by national program priorities. Traditionally, many NARES rice breeding programs followed a single-generation-per-year advancement model, with costs driven predominantly by intensive nursery management and labour-intensive operations, including flowering-stage observations, preharvest evaluations, harvesting, postharvest assessments, and seed processing (**Supplementary Tables S13–S16**).

Preliminary and advanced on-station performance evaluations, typically comprising one- or two-stage annual trials at a single location, revealed variation in trial size and the number of entries assessed. This stage accounted for an average of 6.05% of total pipeline costs, with country-specific allocations: India (6%), Nepal (7.9%), Tanzania (9.4%), and Ghana (2.3%). The key cost drivers included labour, irrigation, and field maintenance, whereas operational efficiency and trial design significantly influenced overall expenditures.

In contrast, Multi-environment Trial (MET) emerged as the most resource-intensive phase, accounting for 45–51% of total breeding pipeline costs, with an average of 47.7% across programs. The scale and complexity of MET vary across countries, shaped by differences in the number of test locations, trial sizes, and experimental designs. The current analysis included the following MET stages: India (SPVT and SUVT), Nepal (CVT1, CVT2, CFFT1, and CFFT2), Tanzania (AYT, MLT1, and MLT2), and Ghana (AYT, MLT1, MLT2, and OFT). Significant investment in MET, ranging from 45 to 51% (49% in India, 51% in Nepal, 45% in Tanzania, and 46% in Ghana), underscores its pivotal role in capturing genotype-by-environment (G×E) interactions to inform varietal selection and advancement. South Asian programs, particularly in India and Nepal, leveraged robust target populations of environments (TPEs) and established partnerships with regional testing networks. In contrast, Ghana and Tanzania faced infrastructural constraints in MET implementation, potentially limiting the representativeness of their TPEs and weakening the precision of selection decisions.

### 3.4 Driving cost and efficiency gains through modernized rice breeding pipelines

**Figure 4:**
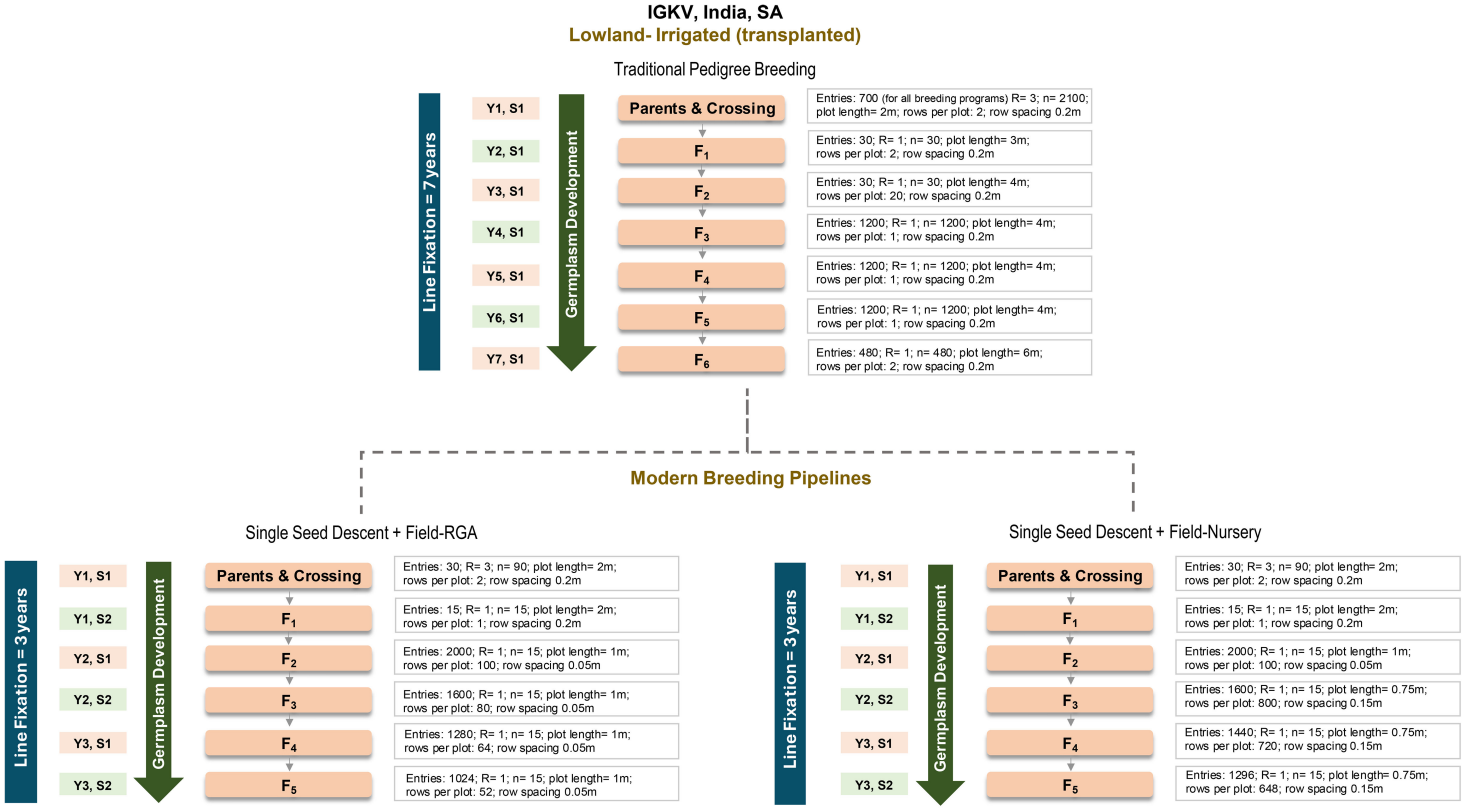
Comparison of traditional and modern rice breeding schemes. Schematic illustration showcasing the accelerated timelines and improved efficiency of modern breeding pipelines; with Field-RGA and Field Nursery, compared to conventional approach, at germplasm development stages and breeding cycle duration.

We analysed the cost efficiencies of three rice breeding pipelines with increasing levels of modernization: (i) conventional pedigree-based breeding, (ii) single-seed descent integrated with field-RGA (SSD+FRGA), and (iii) SSD integrated with field-nursery (SSD+FN) methods (Katiyar et al., 2025). This comparative assessment demonstrated the strategic cost and efficiency gains achieved through the integration of speed breeding technologies within rice pipeline architectures (**Figure 4**). The analysis encompassed the full germplasm development spectrum, from parental selection and crossing through to the development of fixed lines ready for performance evaluation.

A detailed cost analysis revealed significant reductions in expenditure and exceptional gains in efficiency through the adoption of modernized breeding pipelines. The conventional pedigree-based approach incurred USD 7,621.20 to produce just 48 fixed lines from 30 crosses, averaging a mere 1.6 lines per cross. In sharp contrast, pipelines integrating SSD with field-RGA and field-nursery significantly reduced germplasm development costs to USD 3,088.06 and USD 3,940.32, respectively, while delivering 12,285 and 17,490 fixed lines from only 15 crosses, translating to 819 and 1,166 lines per cross. These results highlight the transformative potential of speed breeding technologies in maximizing throughput, minimizing costs, and dramatically improving resource-use efficiency (**Supplementary Table S17****).** The analysis revealed a profound disparity in costs per fixed line, with modern SSD-based pipelines vastly outperforming the conventional approach. SSD integrated with field-RGA, and field-nursery delivered fixed lines at just USD 0.251 and USD 0.225, respectively, compared with an exorbitant USD 158.70 per line under the traditional pedigree-based method, underscoring the cost efficiency of field- based speed breeding platforms (**Supplementary Table S17**). Modernized breeding pipelines also delivered a step-change in spatial efficiency, generating hundreds of fixed lines per cross and drastically reducing land requirements. The conventional pedigree-based approach consumed 6,290.28 sq. m to produce just 1.2 fixed lines per cross, whereas SSD + field-RGA and SSD + field-nursery required only 303 sq. m and 3,908.70 sq. m, respectively, translating to ~20-fold and ~1.6-fold reductions in land use, with several-fold increases in throughput. These findings highlight the superior scalability and land-use optimization of accelerated rice breeding systems.

## 4 Discussion

Accelerating the genetic improvement of rice is essential for ensuring global food security under rising demand. While breeding programs have delivered measurable gains, the pace of progress remains insufficient. Breeding efficiency is typically assessed by the rate of genetic gain per unit time, or the number of varieties released; however, this metric overlooks key constraints, namely, limited time and resources that breeders routinely face. The reported genetic gain for rice yield averages only 0.92%, indicating the scope of acceleration (Seck et al., 2023; Rutkoski and Sparks, 2019a; Rutkoski, 2019b; Li et al., 2018; Bin Rahman and Zhang, 2023). Although many studies report positive trends, they seldom incorporate cost analysis, an omission that limits actionable insights. Embedding cost considerations is vital for evaluating program efficiency and for quantifying the ROI in breeding modernization (Barros et al., 2018; Morais Junior et al., 2017; Atlin and Econopouly, 2022; Katiyar et al., 2025).

This study presents a rigorous cost assessment of conventional rice breeding pipelines in Sub-Saharan Africa and South Asia, which are anchored in four representative NARES breeding programs from Tanzania, Ghana, India, and Nepal. We also quantified the cost-efficiency gains of partially modernized rice breeding pipelines integrating single-seed descent (SSD) with field-RGA and field-nursery (speed breeding technologies). Leveraging the UGCF-Rice, we enabled rigorous cross-program comparisons across diverse institutional and agroecological contexts to identify key cost drivers, reveal inefficiencies, and highlight opportunities for strategic optimization through consistent data capture and robust economic analysis. The framework also facilitates “what-if” simulations to project cost benefits from modifying specific pipeline components without requiring real-world implementation. UGCF- Rice serves multiple strategic functions: (1) enhancing cost transparency through detailed economic profiling of global and national rice breeding pipelines; (2) generating stagewise cost estimates to inform evidence-based investments that improve efficiency, sustainability, and genetic gain; (3) enabling cross-institutional and regional benchmarking; (4) revealing key cost drivers, efficiency gaps, and opportunities through comparisons across ecologies, institutions, and countries; (5) guiding strategic reforms to modernize rice breeding programs; and (6) providing robust, evidence-based data to support sustainable investments and policy development.

### 4.1 Decoding rice breeding costs in South Asia and Sub-Saharan Africa

A clear understanding of the operational design and cost structure of conventional NARES rice breeding pipelines are critical for driving modernization and guiding strategic investment. Our analysis revealed significant variation in pipeline architecture, breeding strategy implementation, alignment with genetic gain principles, and overall cost efficiency. By systematically documenting breeding operations, timelines, scales, and institutional contexts, we established a definitive cost benchmark. Notably, the germplasm development phase exhibited widespread inefficiencies, such as redundant generation advancement (e.g. extended F_6_/F_7_ stages), oversized and poorly prioritized crossing blocks, and suboptimal advancement schemes misaligned with modern breeding principles. Cost disparities were further amplified by differences in trialing scale, stage number, and spatial distribution (e.g. on-station vs. on-farm, single vs. multi-environment trials). Streamlining pipeline design to eliminate redundancies and concentrate resources on stages directly influencing genetic gain and varietal replacement is essential. Strategic breeding scheme optimization, which is grounded in modern breeding theory and cost-benefit analysis, is key to maximizing the return on investment.

### 4.2 Strategic cost analysis as a catalyst for investment and genetic improvement

Conducting a strategic cost analysis of rice breeding in Global South is vital for optimizing investments, improving efficiency, and catalysing engagement from donors and the private sector. Cost insights expose operational bottlenecks, guide technology prioritization, and enable regional benchmarking to fast-track varietal turnover and amplify genetic gains. Comparative analyses highlight how infrastructure, labour, technology, and policy environments shape breeding outcomes, revealing capacity gaps and informing context-specific, cost-effective strategies. Broader costing efforts also shed light on key economic drivers of the rice trade, offering actionable intelligence to advance Africa’s pursuit of rice self-sufficiency.

NARES breeding pipelines are sustained through blended financing, national grants, donor funding (e.g. the CGIAR, BMGF), private investment, and public-private partnerships. Each source carries trade-offs: government funding may be erratic; donor grants are often short-term and administratively demanding; private investment tends to focus on high-return traits; and partnerships require alignment among diverse actors. Strategic integration of these streams is essential to ensure financial resilience and sustained innovation in breeding programs (Das et al., 2025). Sustaining rice breeding performance requires a strategic balance between capital and operational costs. Overinvesting in infrastructure without matching operational support leads to underutilization, whereas excessive operational spending without core assets limits efficiency. Aligning both cost streams with breeding goals, technologies, and timelines is essential to maximize impact and resource use.

Breeding pipelines cost is shaped by program scale, technology use, and location, typically covering crossing, nursery operations, early-generation screening, line fixation, multilocation trials, genotyping, phenotyping, data systems, personnel, infrastructure, and product release. Each stage involves both operational (labour, inputs, services) and capital (infrastructure, equipment, overhead) costs. Accurate, stagewise cost tracking is critical for effective budgeting, efficiency gains, and strategic resource allocation across the breeding pipeline.

### 4.3 Optimized breeding schemes maximize resource efficiency

Optimizing rice breeding pipelines is critical for improving efficiency, reducing costs, and accelerating genetic gain. Key strategies include genomic selection, speed breeding, and data-driven decision tools to refine population size, selection intensity, and cycle duration (Katiyar et al., 2025). Owing to decreasing genotyping costs and the availability of advanced digital tools, selection accuracy and efficiency are rapidly improving. Linking cost per line or cycle to genetic gain enables smarter, outcome-driven investments.

Our analysis shows that pipeline costs vary widely across NARES programs within and between countries, shaped by differences in breeding strategy, breeding scheme optimization, infrastructure, technology access, labour costs, program scale, and agroecological conditions. Reliance on external services, access to genotyping, and participation in regional networks also influence cost structures. Understanding these contextual drivers is essential for equitable investment, effective resource planning, and regional benchmarking. For public-sector breeding programs, optimizing breeding schemes should be a top priority to conserve resources, increase precision, and accelerate varietal development.

### 4.4 Modernizing NARES breeding pipelines for greater efficiency and impact

Measuring and enhancing breeding pipeline efficiency is vital for maximizing the impact of NARES rice programs. Efficiency defines how effectively time, resources, and technologies are translated into improved varieties with measurable genetic gains. It encompasses speed, accuracy, and success across the entire pipeline, starting from crossing to variety release and adoption. Our study demonstrated that strategic, cost-efficient modernization, through shorter cycle durations, lower costs per fixed line, and exponential increases in throughput, significantly boost pipeline performance. These improvements also increase the flexibility of optimizing components of the breeder’s equation, accelerating varietal turnover, annual genetic gain, and the delivery of climate-resilient rice varieties at scale. Marker-assisted selection and genomic selection in combination with predictive breeding require greater initial investments but accelerate breeding cycles and improve selection intensity and selection accuracy, lowering the long-term cost per unit of genetic gain (Nguyen et al., 2023; Juma et al., 2021; Atlin et al., 2017; Cobb et al., 2019). Ultimately, strategies balancing upfront investment with faster, more accurate selection achieve better cost efficiency in terms of genetic gain. The cost per improved rice variety released is a key metric, helping programs evaluate resource use, support strategic planning, and quantify the return on breeding investments.

IGKV in India traditionally relies on conventional pedigree-based breeding pipelines marked by long cycle durations (10–12 years), high resource demands, and low efficiency, averaging only 1.6 fixed lines per cross. A narrow base of fixed lines entering yield trials severely limits selection intensity and hinders genetic gain. This study presents compelling evidence that even incremental modernization through speed breeding using single-seed descent (SSD) combined with field-RGA (F-RGA) and field-nursery (F-N) platforms can dramatically improve the efficiency, scalability, and cost-effectiveness of NARES programs. These field-based, infrastructure-neutral methods leverage dense transplanting under natural conditions to reduce space and inputs, accelerate line fixation, and deliver significant time and resource savings. The true transformative impact of breeding modernization lies in its capacity to exponentially amplify output while simultaneously driving down costs and land use, delivering unparalleled gains in efficiency and scalability.

### 4.5 Strategic pathways to strengthen NARES rice breeding programs globally

Transforming rice breeding in South Asia and Sub-Saharan Africa requires empowering NARES with global science, access to cutting-edge innovative technologies, accelerated breeding modernization frameworks, and best-in-class practices (Katiyar et al., 2025). This empowerment must be built on institutionalized initiatives, transparent costing systems, sustainable resource allocation, and large-scale capacity building. Such a foundation is vital to position NARES as strategic, high-impact partners within the global CGIAR–NARES breeding networks, driving national, regional, and global food and nutrition security. To achieve these goals, the following strategic actions are recommended to strengthen the capacity, efficiency, and impact of NARES rice breeding programs globally: (i) *Institutionalize rigorous, science-driven breeding pipelines*: Develop and implement well-documented, data-informed breeding pipelines, validated through peer review and adaptive feedback, to ensure transparency, scalability, and sustained genetic gain over time. (ii) *Modernize legacy practices through systemic innovation*: Transition from outdated, static processes to dynamic, technology-enabled breeding systems that integrate genomics, precision phenotyping, AI, and predictive analytics. Grounded in quantitative genetics and genomic selection, this transformation must be supported by robust training and comprehensive system upgrades. (iii) *Refocus breeding programs on impact, efficiency, and ROI*: Shift the focus beyond varietal development to prioritize accelerated genetic gain per dollar and per year, emphasizing time-to-market, cost-efficiency, and broad adoption by end users. (iv) *Embed business-oriented models in public breeding programs*: Redesign NARES breeding programs with a business mindset, emphasizing strategic planning, product portfolio optimization, resource prioritization, and measurable impact metrics.

### 4.6 Establishing strategic breeding innovation centres- driving NARES transformation toward SDG 2

**Figure 5:**
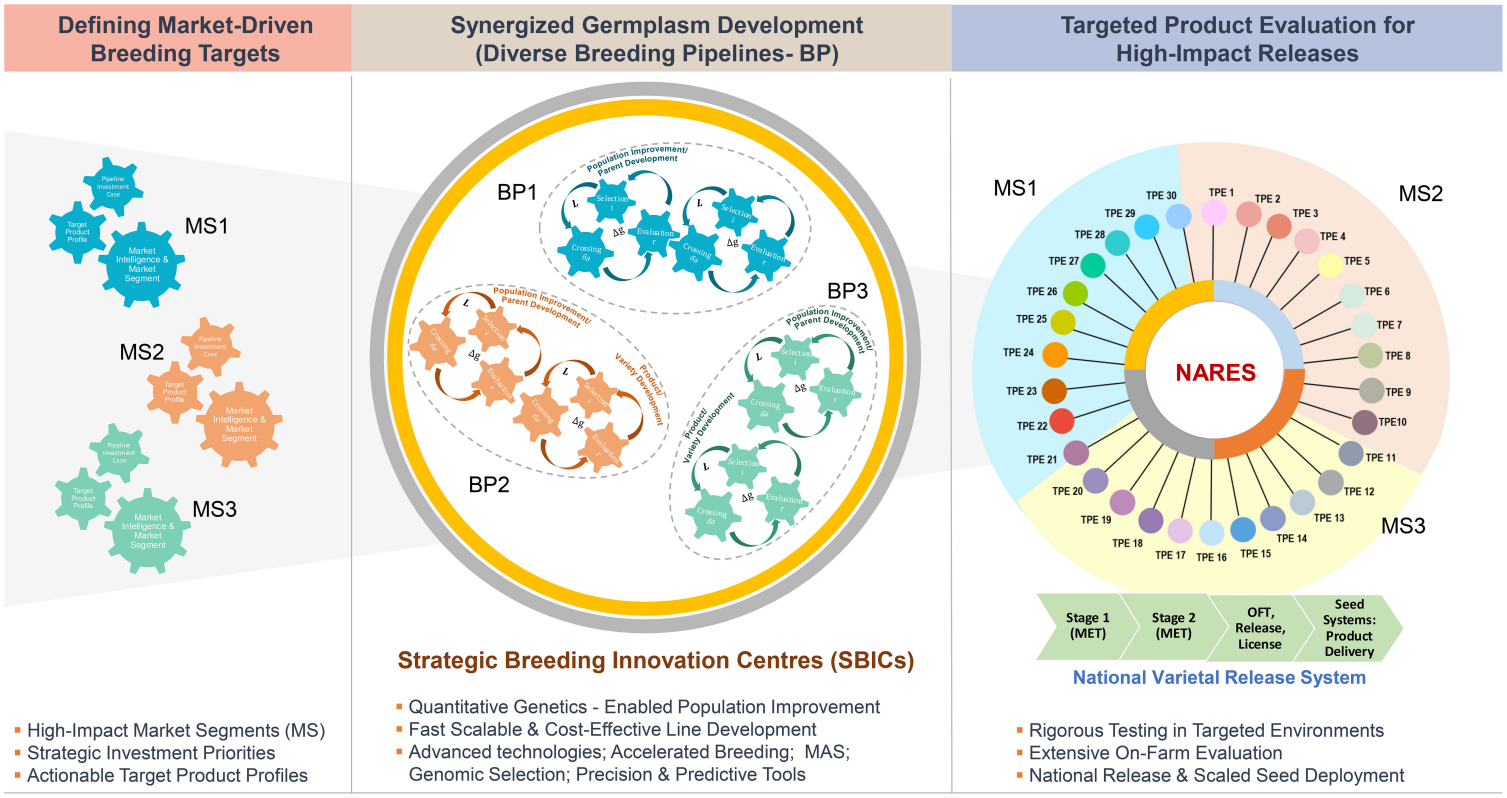
A ‘Cost-Efficient Rice Breeding and Innovation Model’ to empower NARES for global impact. This figure depicts a cost-efficient, integrated rice breeding and innovation model comprising three strategic components: 1. *Market-Driven Breeding Targets*: Defining priority market segments and associated Target Product Profiles (TPPs); 2. *Strategic Breeding Innovation Centres:* centralized facilities driving multiple breeding pipelines through quantitative genetics and cutting-edge tools and technologies with specialized experts; and 3. *Targeted Product Evaluation*: ensuring rigorous testing, on-farm validation, and fast-tracked high impact national release.

Given that not all NARES rice breeding programs can be modernized simultaneously due to limitations in infrastructure, technology access, skilled human resources, market intelligence, quantitative genetics expertise, digital tools, financial capacity, and the required scale of capacity building, there is an urgent need for a pragmatic and scalable solution. With the 2030 SDG 2 deadline approaching fast, we propose an integrated, *“Cost-efficient rice breeding and innovation model to empower NARES for global impact”* (**Figure 5**). This model envisions establishing centralized ‘Strategic Breeding Innovation Centres (SBICs)’, which would be equipped with cutting-edge technologies and expertise, for driving market-informed breeding strategies. The centres would lead quantitative genetic (QG)-based population improvement and facilitate speed/accelerated breeding, marker assisted selection (MAS), genomic selection, predictive breeding, and pipeline optimization. A curated set of elite breeding lines would then be distributed to national programs across countries and target population of environments (TPEs) for rigorous multi-environment testing, selection, and release. This approach would offer a powerful pathway to fast-track genetic gains, optimize resources, and provide all NARES programs with equitable access to innovation, enabling them to deliver high-performing, climate-resilient nutrient-rich rice varieties at a faster pace.

### 4.7 A global framework enabling strategic investment and impact in rice breeding

This study introduces the Unified Global Costing Framework for Rice Breeding (UGCF-Rice), a globally scalable model that fills critical gaps in previous benchmarking efforts, which were often fragmented, localized, and non-comparable due to the lack of a standardized framework. UGCF-Rice in combination with UQ-BPCT enables comprehensive and comparable costing analyses across diverse breeding programs, revealing pronounced cost disparities driven by agroecological diversity, operational scale, and technological maturity across Global South. These insights provide a strong evidence base to guide strategic investments, optimize resource use, shape policies that enhance efficiency, modernization, and long-term sustainability of rice breeding systems worldwide. By establishing clear cost baselines and identifying efficiency gaps, UGCF-Rice equips decision-makers with actionable intelligence to align funding priorities and design sustainable financing strategies that strengthen modernization and resilience. Integrating cost analysis within genetic gain frameworks advances system-level optimization, linking economic efficiency with genetic performance. For breeders and research managers, the findings support evidence-based adoption of cost-effective technologies, operational prioritization, mechanization, automation, and optimization opportunities to improve breeding efficiency. For policymakers and donors, it offers a strong foundation for high-impact, performance-based investments and in establishing strategic alliances such as Strategic Breeding Innovation Centres (SBICs) to transform NARES for global impact. Although the analysis may slightly underestimate costs due to institutional and national program variability, UGCF-Rice establishes the first harmonized model for global cost benchmarking; advancing policy relevance, strategic investment, and operational excellence in rice breeding.

## 5 Conclusions

Cost analysis of rice breeding pipelines is essential to ensure every research dollar accelerates genetic gains and delivers climate-resilient varieties, especially in an era of shrinking R&D budgets and escalating global food insecurity. This study introduces the first Unified Global Costing Framework for Rice Breeding (UGCF-Rice) to benchmark rice breeding costs, showcases its global scalability through case studies in India, Nepal, Ghana, and Tanzania, and establishes a data-driven foundation for modernizing breeding systems. Using the UGCF-Rice in combination with UQ-BPCT, it identified key cost drivers, efficiency gaps, and strategic opportunities for optimization. The framework generates robust, comparable cost metrics to guide resource allocation, investment prioritization, and cross-program benchmarking. Integrating speed breeding technologies with single-seed descent (SSD) proved transformative, reducing cycle time by up to 2.3-fold, increasing throughput 17–24 times, and lowering land use 1.6–20 times without additional cost. These results demonstrate that breeding modernization is essential to enhancing cost-efficiency, maximizing return on investment, and accelerating genetic gains.

Building on these insights, the proposed ‘Cost-efficient Rice Breeding and Innovation Model’ provides a strategic pathway to transform NARES breeding programs into agile, data-driven, and performance-oriented systems through Strategic Breeding Innovation Centres (SBICs). By institutionalizing cost transparency and efficiency-based planning, this model empowers NARES to achieve faster varietal turnover, strengthen food systems, and amplify global breeding impact, positioning the Global South as a driving force for future-ready, sustainable rice innovation.

## Data Availability

The original contributions presented in the study are included in the article/**Supplementary Material**. Further inquiries can be directed to the corresponding author.

## Glossary

ABI: Accelerated Breeding Initiative
AICRIP: All India Coordinated Rice Improvement Project
ANOVA: Analysis of Variance
AVT: Advanced Varietal Trials
AYT: Advanced Yield Trial
BM: Breeding Modernization
BMGF: Bill & Melinda Gates Foundation
CD: Critical Difference
CFFT: Coordinated Farmers' Field Trial
CGIAR: Consultative Group on International Agricultural Research
CRI: CSIR-Crops Research Institute
CV: Coefficient of Variation
CVT: Coordinated Varietal Trial
DUS: Distinctiveness, Uniformity, and Stability
FAT: Final Advanced Trial
G×E: Genotype-by-environment
GS: Genomic Selection
IGKV: Indira Gandhi Krishi Vishwavidyalaya
IVT: Initial Varietal Trials
MAS: Marker Assisted Selection
MET: Multi- environment Trial
MLT: Multilocation Trial
NARC: Nepal Agricultural Research Council
NARES: National Agricultural Research and Extension System
NPT: National Performance Trial
NRRP: National Rice Research Program
NVRRC: National Variety Release and Registration Committee
OFT: On-Farm Trial
P&C: Parental Selection and Crossing
QG: Quantitative Genetics
R&D: Research and Development
RGA: Rapid Generation Advancement
ROI: Return on Investment
SA: South Asia
SBICs: Strategic Breeding Innovation Centres
SDGs: Sustainable Development Goals
SED: Standard Error of Difference
SIET: Station Initial Evaluation Trail
SPVT: State Preliminary Varietal Trial
SSA: Sub-Saharan Africa
SSD: Single-Seed Descent
SUVT: State Uniform Varietal Trial
TARI: Tanzania Agricultural Research Institute
TOSCI: Tanzania Official Seed Certification Institute
TPEs: Target Population of Environments
UGCF-Rice: Unified Global Costing Framework for Rice Breeding
UQ-BPCT: University of Queensland’s Breeding Program Costing Tool
