# Supplementary material for: A unified global costing framework catalyzes strategic investment in rice breeding
Source: Front Plant Sci. 2026 Jan 16;16:1681605. doi: 10.3389/fpls.2025.1681605 (PMC12855492; doi:10.3389/fpls.2025.1681605)
Supplement: Supplementary file 2 [file Table2.docx]

**Table S7.**  **UGCF-Rice Module 3: Breeding Operations and Activities Across the Pipeline Stages**

Crop: Rice

Institute:

Market Segment:

Breeding Program:

Breeding Pipeline:

| **Pre-sowing operations: Germplasm Development and Testing & Trialing- Common activities** | | | | | | | | |
| --- | --- | --- | --- | --- | --- | --- | --- | --- |
| ***Activity*** | ***Sub-Activity*** | ***Cost type (Input/Labour)*** | ***Unit Cost (Currency)*** | ***Comments*** | | ***Cost Per Acre/Hectare*** | ***No. of Times (Frequency of the activity)*** |  |
| **Land Preparation (LP)** | **XA1- LP- Ploughing 1 -Pre-season** |  |  |  | |  |  |  |
| ***Cost of Land Preparation- LP- Ploughing 1 -Pre-season- per m2*** |  |  |  |  | |  |  |  |
| *XA1 Cost of fuel for pre-season ploughing per m2* |  |  |  |  | |  |  |  |
| *XA1-Labour cost of LP- Ploughing 1 -Pre-season- per m2* |  |  |  |  | |  |  |  |
| **Land Preparation (LP)** | **XA2- LP- Ploughing 2 -Nursery** |  |  |  | |  |  |  |
| *XA2-Labour cost of LP- Ploughing 2 -Nursery per m2* |  |  |  |  | |  |  |  |
| **Land Preparation (LP)** | **XA3- LP- Ploughing 3 -Transplanting** |  |  |  | |  |  |  |
| *XA3-Labour cost of LP- Ploughing 3 -Transplanting per m2* |  |  |  |  | |  |  |  |
| **Land Preparation (LP)** | **XA4- LP- Field Cleaning (Manual)** |  |  |  | |  |  |  |
| *XA4 Labour cost of LP- Field Cleaning (Manual) per m2* |  |  |  |  | |  |  |  |
| **Land Preparation (LP)** | **XA5- LP- Rotavator + Land leveling** |  |  |  | |  |  |  |
| *XA5 Labour cost of LP- Rotavator + Land leveling per m2* |  |  |  |  | |  |  |  |
| **Land Preparation (LP)** | **XA6- LP- Bunding** |  |  |  | |  |  |  |
| *XA6 Labour cost of LP- Bunding per m2* |  |  |  |  | |  |  |  |
| **Land Preparation (LP)** | **XA7- LP- Canal Cleaning** |  |  |  | |  |  |  |
| *XA7 Labour cost of LP- Canal Cleaning per m2* |  |  |  |  | |  |  |  |
| **Seed Preparation (SP)** | **XB1- SP- Seed Sorting** |  |  |  | |  |  |  |
| *XB1 Cost of Paper Envelop per unit* |  |  |  |  | |  |  |  |
| *XB1 Labour cost of SP- Seed Sorting per m2* |  |  |  |  | |  |  |  |
| **Seed Preparation (SP)** | **XB2- SP- Seed Packet labeling** |  |  |  | |  |  |  |
| *XB2 Cost of Seed Packet Label - SP- Seed Packet labeling per unit* |  |  |  |  | |  |  |  |
| *XB2 Labour cost of SP- Seed Packet labeling per m2* |  |  |  |  | |  |  |  |
| **Seed Preparation (SP)** | **XB3- SP- Seed Treatment** |  |  |  | |  |  |  |
| *XB3 Cost of Fungicide- SP- Seed Treatment per unit* |  |  |  |  | |  |  |  |
| *XB3 Labour cost of SP- Seed Treatment per m2* |  |  |  |  | |  |  |  |
| **Seed Preparation (SP)** | **XB4- SP- Seed Transportation** |  |  |  | |  |  |  |
| *XB4 Cost of Courier packaging & dispatch for MLTs per location* |  |  |  |  | |  |  |  |
| *XB4 Labour cost of SP- Seed Transportation per m2* |  |  |  |  | |  |  |  |
| **Nursery/Seedlings** |  |  |  |  | |  |  |  |
| ***Activity*** | ***Sub-Activity*** | ***Cost type (Input/Labour)*** | ***Unit Cost (Currency)*** | ***Comments*** | | ***Cost Per Acre/Hectare*** | ***No. of Times*** |  |
| **Nursery/ Seedlings - (GD + T&T)** |  |  |  |  | |  |  |  |
| *XC1 Labour cost of Nursery - Bed Preparation per m2* |  |  |  |  | |  |  |  |
| **Nursery / Seedlings - (GD + T&T)** |  |  |  |  | |  |  |  |
| *XC2 Labour cost of Nursery - Rows Preparation per m2* |  |  |  |  | |  |  |  |
| **Nursery/ Seedlings - (GD + T&T)** | **XC3- Nursery - Sowing** |  |  |  | |  |  |  |
| *XC3 Labour cost of Nursery - Sowing per m2* |  |  |  |  | |  |  |  |
| **Nursery/ Seedlings - (GD + T&T)** | **XC4- Nursery - Labeling** |  |  |  | |  |  |  |
| *Cost of Nursery/ Seedlings- Nursery labelling* |  |  |  |  | |  |  |  |
| *XC4 Cost of bamboo stick -Nursery labelling per unit* |  |  |  |  | |  |  |  |
| *XC4 Cost of Field Label - Nursery labelling per unit* |  |  |  |  | |  |  |  |
| *XC4 Labour cost of Nursery labelling per m2* |  |  |  |  | |  |  |  |
| **Transplanting** |  |  |  |  | |  |  |  |
| ***Activity*** | ***Sub-Activity*** | ***Cost type (Input/Labour)*** | ***Unit Cost (Currency)*** | ***Comments*** | | ***Cost Per Acre/Hectare*** | ***No. of Times*** |  |
| ***Transplanting*** | **XD1- Transplanting - Layout** |  |  |  | |  |  |  |
| *XD1 Labour cost of Transplanting- Layout per m2* |  |  |  |  | |  |  |  |
| **Transplanting** | **XD2- Transplanting - Nursery Uprooting** |  |  |  | |  |  |  |
| *XD2 Labour cost of Transplanting-Nursery Uprooting per m2* |  |  |  |  | |  |  |  |
| **Transplanting** | **XD3- Transplanting - Nursery Transport** |  |  |  | |  |  |  |
| *XD3 Labour cost of Transplanting- Nursery transport per m2* |  |  |  |  | |  |  |  |
| **Transplanting** | **XD4- Transplanting - Nursery Distribution** |  |  |  | |  |  |  |
| *XD4 Labour cost of Transplanting- Nursery distribution per m2* |  |  |  |  | |  |  |  |
| **Transplanting** | **XD5- Transplanting - Transplanting** |  |  |  | |  |  |  |
| *XD5 Labour cost of Transplanting (Normal 20 cm X 20 cm) per m2* |  |  |  |  | |  |  |  |
| **Transplanting** | **XD6- Transplanting - Gap Filling** |  |  |  | |  |  |  |
| *XD6 Labour cost of Transplanting-Gap filling per m2* |  |  |  |  | |  |  |  |
| **Agricultural Inputs & Management** |  |  |  |  | |  |  |  |
| ***Activity*** | ***Sub-Activity*** | ***Cost type (Input/Labour)*** | ***Unit Cost (Currency)*** | ***Comments*** | | ***Cost Per Acre/Hectare*** | ***No. of Times*** |  |
| **Agricultural Inputs & Management (AIM)** | **XE1- AIM- Irrigation** |  |  |  | |  |  |  |
| ***Cost of AIM- Irrigation per m2*** |  |  |  |  | |  |  |  |
| *XE1 Cost of Electricity - AIM- Irrigation per m2* |  |  |  |  | |  |  |  |
| *XE1 Labour cost of AIM- Irrigation per m2* |  |  |  |  | |  |  |  |
| **Agricultural Inputs & Management (AIM)** | **XE2- AIM- Fertilizer application** |  |  |  | |  |  |  |
| ***Cost of AIM- Fertilizer application per m2*** |  |  |  |  | |  |  |  |
| *XE2 Cost of Fertilizer - DAP- AIM- Fertilizer application per m2* |  |  |  |  | |  |  |  |
| *XE2 Cost of Fertilizer - Potassium- AIM- Fertilizer application per m2* |  |  |  |  | |  |  |  |
| *XE2 Cost of Fertilizer - Urea- AIM- Fertilizer application per m2* |  |  |  |  | |  |  |  |
| *XE2 Labour cost of AIM- Fertilizer application per m2* |  |  |  |  | |  |  |  |
| **Agricultural Inputs & Management (AIM)** | **XE3- AIM- Herbicide (pre-emergence)** |  |  |  | |  |  |  |
| ***Cost of AIM- Herbicide (pre-emergence) per m2*** |  |  |  |  | |  |  |  |
| *XE3 Cost of Herbicide-2,4-D- AIM- Herbicide (pre-emergence) per m2* |  |  |  |  | |  |  |  |
| *XE3 Cost of Herbicide-Glyphosate- AIM- Herbicide (pre-emergence) per m2* |  |  |  |  | |  |  |  |
| *XE3 Labour cost of AIM- Herbicide (pre-emergence) per m2* |  |  |  |  | |  |  |  |
| **Agricultural Inputs & Management (AIM)** | **XE4- AIM- Herbicide (post-emergence)** |  |  |  | |  |  |  |
| ***Cost of AIM- Post-emergence herbicide application per m2*** |  |  |  |  | |  |  |  |
| *XE4 Cost of Nomoni Gold- AIM- Herbicide (post-emergence) per m2* |  |  |  |  | |  |  |  |
| *XE4 Labour cost of AIM- Post-emergence herbicide application per m2* |  |  |  |  | |  |  |  |
| **Agricultural Inputs & Management (AIM)** | **XE5- AIM- Manual Bund cleaning** |  |  |  | |  |  |  |
| *XE5 Labour cost of AIM- Manual Bund cleaning per m2* |  |  |  |  | |  |  |  |
| **Agricultural Inputs & Management (AIM)** | **XE6- AIM- Rodenticides** |  |  |  | |  |  |  |
| ***Cost of AIM- Rodenticides application per m2*** |  |  |  |  | |  |  |  |
| *XE6 Cost of Rodenticide- AIM- Rodenticides per m2* |  |  |  |  | |  |  |  |
| *XE6 Labour cost of AIM- Rodenticides application per m2* |  |  |  |  | |  |  |  |
| **Agricultural Inputs & Management (AIM)** | **XE7- AIM- Insecticide** |  |  |  | |  |  |  |
| ***Cost of AIM- Insecticide per m2*** |  |  |  |  | |  |  |  |
| *XE7 Cost of Insecticide- AIM- Insecticide per m2* |  |  |  |  | |  |  |  |
| *XE7 Labour cost of AIM- Insecticide per m2* |  |  |  |  | |  |  |  |
| **Agricultural Inputs & Management (AIM)** | **XE8- AIM- Fungicide** |  |  |  | |  |  |  |
| ***Cost of AIM- Fungicide per m2*** |  |  |  |  | |  |  |  |
| *XE8 Cost of Fungicide- AIM- Fungicide per m2* |  |  |  |  | |  |  |  |
| *XE8 Labour cost of AIM- Fungicide per m2* |  |  |  |  | |  |  |  |
| **Agricultural Inputs & Management (AIM)** | **XE9- AIM- Manual weeding** |  |  |  | |  |  |  |
| *XE9 Labour cost of AIM- Manual weeding per m2* |  |  |  |  | |  |  |  |
| **Agricultural Inputs & Management (AIM)** | **XE10- AIM- Bird scarer** |  |  |  | |  |  |  |
| *XE10 Labour cost of AIM- Bird scarer per m2* |  |  |  |  | |  |  |  |
| **Agricultural Inputs & Management (AIM)** | **XE11- AIM- Transportation** |  |  |  | |  |  |  |
| *XE11 Cost of fuel - AIM- Transportation per trip* |  |  |  |  | |  |  |  |
| *XE11 Labour Cost - AIM- Transportation per trip* |  |  |  |  | |  |  |  |
| **Stationary & miscellaneous Items** | **XF1- Stationary & miscellaneous item** |  |  |  | |  |  |  |
| *XF1 Cost of Field Book per unit* |  |  |  |  | |  |  |  |
| *XF1 Cost of Marker pen per unit* |  |  |  |  | |  |  |  |
| *XF1 Cost of Pencil per unit* |  |  |  |  | |  |  |  |
| *XF1 Cost of Ballpen per unit* |  |  |  |  | |  |  |  |
| *XF1 Cost of Sharpener per unit* |  |  |  |  | |  |  |  |
| *XF1 Cost of Ruler per unit* |  |  |  |  | |  |  |  |
| *XF1 Cost of meter scale per unit* |  |  |  |  | |  |  |  |
| *XF1 Cost of Eraser per unit* |  |  |  |  | |  |  |  |
| *XF1 Cost of Stapler pin per unit* |  |  |  |  | |  |  |  |
| *XF1 Cost of Stapler per unit* |  |  |  |  | |  |  |  |
| *XF1 Cost of Measuring tape per unit* |  |  |  |  | |  |  |  |
| *XF1 Cost of Cap per unit* |  |  |  |  | |  |  |  |
| *XF1 Cost of Laser printer toner per unit* |  |  |  |  | |  |  |  |
| *XF1 Cost of A4 sized Paper per unit* |  |  |  |  | |  |  |  |
| *XF1 Cost of Graph paper per unit* |  |  |  |  | |  |  |  |
| *XF1 Cost of Rubber band per unit* |  |  |  |  | |  |  |  |
| *XF1 Cost of Calculator per unit* |  |  |  |  | |  |  |  |
| *XF1 Cost of Torch light per unit* |  |  |  |  | |  |  |  |
| *XF1 Cost of Measuring cylinders (1000ml)- AIM- Fertilizer application per unit* |  |  |  |  | |  |  |  |
| *XF1 Cost of First-aid kit- AIM- Herbicide (pre-emergence) per unit* |  |  |  |  | |  |  |  |
| *Total Cost of all Stationary & Miscellaneous Items per m2* |  |  |  |  | |  |  |  |
| *XF1 Cost of Stationary & Miscellaneous Items per m2* |  |  |  |  | |  |  |  |
|  |  |  |  |  | |  |  |  |
| **Germplasm Development- operations & activities** |  |  |  |  | |  |  |  |
| **Parents & Crossing** |  |  |  |  | |  |  |  |
| ***Activity*** | ***Sub-Activity*** | ***Cost type (Input/Labour)*** | ***Unit Cost (Currency)*** | ***Comments*** | | ***Cost Per Acre/Hectare*** | ***No. of Times*** |  |
| **YA1 Crossing- Parents & Hybridization** | **YA1- Crossing- Hybridization** |  |  |  | |  |  |  |
| ***Cost of Crossing- Hybridization*** |  |  |  |  | |  |  |  |
| *YA1 Cost of U-pin- Crossing- Hybridization per unit* |  |  |  |  | |  |  |  |
| *YA1 Cost of Electric Bulbs- Crossing- Hybridization per unit* |  |  |  |  | |  |  |  |
| *YA1 Cost of Butter Paper bags- Crossing- Hybridization per unit* |  |  |  |  | |  |  |  |
| *YA1 Cost of Bucket (5 lt)- P- Sowing per trial* |  |  |  |  | |  |  |  |
| *YA1 Labour cost of Parent- Seed sowing & labeling in buckets* |  |  |  |  | |  |  |  |
| *YA1 Labour cost of Crossing- Hybridization per cross* |  |  |  |  | |  |  |  |
| **YA2 P&C- Harvesting** | **YA2-P&C- Harvesting** |  |  |  | |  |  |  |
| *YA2 Cost of Harvesting bag (2kg)- P&C- Harvesting per unit* |  |  |  |  | |  |  |  |
| *YA2 Cost of Field Labels- P&C- Harvesting per unit* |  |  |  |  | |  |  |  |
| *YA2 Cost of Paper envelops- P&C-Harvesting per unit* |  |  |  |  | |  |  |  |
| *YA2 Labour cost of P&C- Harvesting per m2* |  |  |  |  | |  |  |  |
| **YA3 P&C- Seed processing** | **YA3-P&C- Seed processing** |  |  |  | |  |  |  |
| ***Cost of P&C- Seed processing per m2*** |  |  |  |  | |  |  |  |
| *YA3 Cost of Seed Packet Labels-P&C- Seed processing per unit* |  |  |  |  | |  |  |  |
| *YA3 Labour cost of P&C- Seed processing per m2* |  |  |  |  | |  |  |  |
|  |  |  |  |  | |  |  |  |
| **F1s** |  |  |  |  | |  |  |  |
| **F1s- Nursery/ Seedlings** | **YB1- F1s- Sowing** |  |  |  | |  |  |  |
| ***Cost of F1s- Sowing in buckets per m2*** |  |  |  |  | |  |  |  |
| *YB1 Cost of Bucket (5 lt)- F1s- Sowing per unit* |  |  |  |  | |  |  |  |
| *YB1 Cost of disposable petri plate- F1s- Sowing per unit* |  |  |  |  | |  |  |  |
| *YB1 Labour cost of F1s- Sowing in buckets* |  |  |  |  | |  |  |  |
| **F1s- Nursery/ Seedlings** | **YB2- F1s- Labeling** |  |  |  | |  |  |  |
| **Cost of F1s- Nursery/ Seedlings- F1s- Labeling per m2** |  |  |  |  | |  |  |  |
| *YB2 Cost of Bamboo stick -F1s- Labeling per unit* |  |  |  |  | |  |  |  |
| *YB2 Cost of Field Label - F1s- Labeling per unit* |  |  |  |  | |  |  |  |
| *YB2 Labour cost of F1s- Labeling per m2* |  |  |  |  | |  |  |  |
| **F1s- Nursery/ Seedlings** | **YB3- F1s- Harvesting** |  |  |  | |  |  |  |
| *YB3 Cost of F1s paper envelops- F1s-Harvesting per unit* |  |  |  |  | |  |  |  |
| *YB3 Labour cost of F1- Harvesting per m2* |  |  |  |  | |  |  |  |
| **F1s- Nursery/ Seedlings** | **YB4- F1 derived F2 Seed Threshing & processing** |  |  |  | |  |  |  |
| *YB4 Cost of Seed Packet Label-F1s- Seed processing per unit* |  |  |  |  | |  |  |  |
| *YB4 Cost of Paper envelop-F1s-Seed processing per unit* |  |  |  |  | |  |  |  |
| *YB4 Labour cost of F1 derived F2 Seed processing per m2* |  |  |  |  | |  |  |  |
|  |  |  |  |  | |  |  |  |
| **F2** |  |  |  |  | |  |  |  |
| **F2- Observations at Flowering** | **YC1- F2- Observations at Flowering** |  |  |  | |  |  |  |
| *YC1 Labour cost of F2- Observations at Flowering per m2* |  |  |  |  | |  |  |  |
| **F2- Observations at Pre-harvest** | **YC2- F2- Observations at Pre-harvest** |  |  |  | |  |  |  |
| *YC2 Labour cost for F2- Observations at Pre-harvest per m2* |  |  |  |  | |  |  |  |
| **F2- Harvesting** | **YC3- F2- Harvesting** |  |  |  | |  |  |  |
| *YC3 Cost of Harvesting bag (2kg)- F2- Harvesting & Seed processing per unit* |  |  |  |  | |  |  |  |
| *YC3 Labour cost of F2- Harvesting per m2* |  |  |  |  | |  |  |  |
| **F2- Seed processing** | **YC4- F2- Seed processing** |  |  |  | |  |  |  |
| *YC4 Cost of Gunny bag- F2-Seed processing per unit* |  |  |  |  | |  |  |  |
| *YC4 Labour cost of F2- Seed processing per m2* |  |  |  |  | |  |  |  |
|  |  |  |  |  | |  |  |  |
| **F3** |  |  |  |  | |  |  |  |
| **F3- Observations at Flowering** | **YD1- F3- Observations at Flowering** |  |  |  | |  |  |  |
| *YD1 Labour cost of F3- Observations at Flowering per m2* |  |  |  |  | |  |  |  |
| **F3- Observations at Pre-harvest** | **YD2- F3- Observations at Pre-harvest** |  |  |  | |  |  |  |
| *YD2 Labour cost of F3- Observations at Pre-harvest per m2* |  |  |  |  | |  |  |  |
| **F3- Harvesting** | **YD3- F3- Harvesting** |  |  |  | |  |  |  |
| *YD3 Cost of Harvesting bag (2kg)- F3- Harvesting & Seed processing per unit* |  |  |  |  | |  |  |  |
| *YD3 Labour cost of F3- Harvesting per m2* |  |  |  |  | |  |  |  |
| **F3- Seed processing** | **YD4- F3- Seed processing** |  |  |  | |  |  |  |
| *YD4 Cost of Gunny bag- F3-Seed processing per unit* |  |  |  |  | |  |  |  |
| *YD4 Labour cost of F3- Seed processing per m2* |  |  |  |  | |  |  |  |
|  |  |  |  |  | |  |  |  |
| **F4** |  |  |  |  | |  |  |  |
| **F4- Observations at Flowering** | **YE1- F4- Observations at Flowering** |  |  |  | |  |  |  |
| *YE1 Labour cost of F4- Observations at Flowering per m2* |  |  |  |  | |  |  |  |
| **F4- Observations at Pre-harvest** | **YE2- F4- Observations at Pre-harvest** |  |  |  | |  |  |  |
| *YE2 Labour cost of F4 -Observations at Pre-harvest per m2* |  |  |  |  | |  |  |  |
| **F4- Harvesting** | **YE3- F4- Harvesting** |  |  |  | |  |  |  |
| *YE3 Cost of Harvesting bag (2kg)- F4- Harvesting & Seed processing per unit* |  |  |  |  | |  |  |  |
| *YE3 Labour cost of F4- Harvesting per m2* |  |  |  |  | |  |  |  |
| **F4- Seed processing** | **YE4- F4- Seed processing** |  |  |  | |  |  |  |
| *YE4 Cost of Gunny bag- F4-Seed processing per unit* |  |  |  |  | |  |  |  |
| *YE4 Labour cost of F4- Seed processing per m2* |  |  |  |  | |  |  |  |
|  |  |  |  |  | |  |  |  |
| **F5** |  |  |  |  | |  |  |  |
| **F5- Observations at Flowering** | **YF1- F5- Observations at Flowering** |  |  |  | |  |  |  |
| *YF1 Labour cost of F5- Observations at Flowering per m2* |  |  |  |  | |  |  |  |
| **F5- Observations at Pre-harvest** | **YF2- F5- Observations at Pre-harvest** |  |  |  | |  |  |  |
| *YF2 Labour cost of F5- Observations at Pre-harvest per m2* |  |  |  |  | |  |  |  |
| **F5- Harvesting** | **YF3- F5- Harvesting** |  |  |  | |  |  |  |
| *YF3 Cost of Harvesting bag (2kg)- F5- Harvesting & Seed processing per unit* |  |  |  |  | |  |  |  |
| *YF3 Labour cost of F5- Harvesting per m2* |  |  |  |  | |  |  |  |
| **F5- Seed processing** | **YF4- F5- Seed processing** |  |  |  | |  |  |  |
| *YF4 Cost of Gunny bag- F5-Seed processing per unit* |  |  |  |  | |  |  |  |
| *YF4 Labour cost of F5- Seed processing per m2* |  |  |  |  | |  |  |  |
|  |  |  |  |  | |  |  |  |
| **F6** |  |  |  |  | |  |  |  |
| **F6- Observations at Flowering** | **YG1- F6- Observations at Flowering** |  |  |  | |  |  |  |
| *YG1 Labour cost of F6- Observations at Flowering per m2* |  |  |  |  | |  |  |  |
| **F6- Observations at Pre-harvest** | **YG2- F6- Observations at Pre-harvest** |  |  |  | |  |  |  |
| *YG2 Labour cost of F6- Observations at Pre-harvest per m2* |  |  |  |  | |  |  |  |
| **F6- Harvesting** | **YG3- F6- Harvesting** |  |  |  | |  |  |  |
| *YG3 Cost of Harvesting bag (2kg)- F6- Harvesting & Seed processing per unit* |  |  |  |  | |  |  |  |
| *YG3 Labour cost of F6- Harvesting per m2* |  |  |  |  | |  |  |  |
| **F6- Seed processing** | **YG4- F6- Seed processing** |  |  |  | |  |  |  |
| *YG4 Cost of Gunny bag- F6-Seed processing per unit* |  |  |  |  | |  |  |  |
| *YG4 Labour cost of F6- Seed processing per m2* |  |  |  |  | |  |  |  |
| **F6- Post-harvest Observations** | **YG5- Post-harvest Observations** |  |  |  | |  |  |  |
| ***Cost of F6- Post-harvest Observations per m2*** |  |  |  |  | |  |  |  |
| *YG5 Cost of Field Label- F6- Post-harvest Observations per unit* |  |  |  |  | |  |  |  |
| *YG5 Cost of Paper envelop- F6- Post-harvest Observations per unit* |  |  |  |  | |  |  |  |
| *YG5 Labour cost of F6- Post-harvest Observations per m2* |  |  |  |  | |  |  |  |
|  |  |  |  |  | |  |  |  |
| **Testing & Trialing - operations & activities** |  |  |  |  | |  |  |  |
| **Stage 1 trial** |  |  |  |  | |  |  |  |
| **Stage 1 trial- Observations at Flowering** | **ZA1- Stage 1 trial- Observations at Flowering** |  |  |  | |  |  |  |
| *ZA1 Labour cost of Stage 1 trial- Observations at Flowering per m2* |  |  |  |  | |  |  |  |
| **Stage 1 trial- Observations at Pre-harvest** | **ZA2- Stage 1 trial- Observations at Pre-harvest** |  |  |  | |  |  |  |
| *ZA2 Labour cost of Stage 1 trial- Observations at Pre-harvest per m2* |  |  |  |  | |  |  |  |
| **Stage 1 trial- Harvesting** | **ZA3- Stage 1 trial- Harvesting** |  |  |  | |  |  |  |
| *ZA3 Labour cost of Stage 1 trial- Harvesting per m2* |  |  |  |  | |  |  |  |
| **Stage 1 trial- Seed processing** | **ZA4- Stage 1 trial- Seed processing** |  |  |  | |  |  |  |
| *ZA4 Cost of Cloth bag- Stage 1 trial-Harvesting & Seed processing per unit* |  |  |  |  | |  |  |  |
| *ZA4 Cost of Gunny bag- Stage 1 trial-Seed processing per unit* |  |  |  |  | |  |  |  |
| *ZA4 Labour cost of Stage 1 trial- Seed processing per m2* |  |  |  |  | |  |  |  |
| **Stage 1 trial- Post-harvest Observations** | **ZA5- Stage 1 trial- Post-harvest Observations** |  |  |  | |  |  |  |
| *ZA5 Cost of Seed Packet Label- Stage 1 trial- Post-harvest Observations per unit* |  |  |  |  | |  |  |  |
| *ZA5 Cost of Paper envelop- Stage 1 trial- Post-harvest Observations per unit* |  |  |  |  | |  |  |  |
| *ZA5 Labour cost of Stage 1 trial- Post-harvest Observations per m2* |  |  |  |  | |  |  |  |
|  |  |  |  |  | |  |  |  |
| **Stage 2 trial** |  |  |  |  | |  |  |  |
| **Stage 2 trial- Observations at Flowering** | **ZB1- Stage 2 trial- Observations at Flowering** |  |  |  | |  |  |  |
| *ZB1 Labour cost of Stage 2 trial Observations at Flowering per m2* |  |  |  |  | |  |  |  |
| **Stage 2 trial- Observations at Pre-harvest** | **ZB2- Stage 2 trial- Observations at Pre-harvest** |  |  |  | |  |  |  |
| *ZB2 Labour cost of Stage 2 trial- Observations at Pre-harvest per m2* |  |  |  |  | |  |  |  |
| **Stage 2 trial- Harvesting** | **ZB3- Stage 2 trial- Harvesting** |  |  |  | |  |  |  |
| *ZB3 Labour cost of Stage 2 trial- Harvesting per m2* |  |  |  |  | |  |  |  |
| **Stage 2 trial- Seed processing** | **ZB4- Stage 2 trial- Seed processing** |  |  |  | |  |  |  |
| *ZB4 Cost of Cloth bag- Stage 2 trial-Harvesting & Seed processing per unit* |  |  |  |  | |  |  |  |
| *ZB4 Cost of Gunny bag- Stage 2 trial-Seed processing per unit* |  |  |  |  | |  |  |  |
| *ZB4 Labour cost of Stage 2 trial- Seed processing per m2* |  |  |  |  | |  |  |  |
| **Stage 2 trial- Post-harvest Observations** | **ZB5- Stage 2 trial- Post-harvest Observations** |  |  |  | |  |  |  |
| *ZB5 Cost of Seed Packet Label- Stage 2 trial- Post-harvest Observations per unit* |  |  |  |  | |  |  |  |
| *ZB5 Cost of Paper envelop- Stage 2 trial- Post-harvest Observations per unit* |  |  |  |  | |  |  |  |
| *ZB5 Labour cost of Stage 2 trial- Post-harvest Observations per m2* |  |  |  |  | |  |  |  |
|  |  |  |  |  | |  |  |  |
| **Stage 3 trial** |  |  |  |  | |  |  |  |
| *Stage 3 trial- Cost of Fuel for monitoring trial- Site visit per trip* |  |  |  |  | |  |  |  |
| *Stage 3 trial- Cost of Fuel for monitoring trial- Site visit per trip* |  |  |  |  | |  |  |  |
| *Stage 3 trial- Cost of Fuel for monitoring trial- Site visit per trip* |  |  |  |  | |  |  |  |
| *Stage 3 trial- Cost of Land lease - Site per m2* |  |  |  |  | |  |  |  |
| *Stage 3 trial- Cost of Land lease - Site per m2* |  |  |  |  | |  |  |  |
| *Stage 3 trial- Cost of Land lease - Site per m2* |  |  |  |  | |  |  |  |
| *1 Breeder per diem per day-outstation* |  |  |  |  | |  |  |  |
| *1 Field technician per diem per day-outstation* |  |  |  |  | |  |  |  |
| *1 Driver per diem per day-outstation* |  |  |  |  | |  |  |  |
| **Stage 3 trial - Observations at Flowering** | **ZC1- Stage 3 trial- Observations at Flowering** |  |  |  | |  |  |  |
| *ZC1 Labour cost of Stage 3 trial -Observations at Flowering per m2* |  |  |  |  | |  |  |  |
| **Stage 3 trial - Observations at Pre-harvest** | **ZC2- Stage 3 trial- Observations at Pre-harvest** |  |  |  | |  |  |  |
| *ZC2 Labour cost at Harvesting- Stage 3 trial - Observations at Pre-harvest per m2* |  |  |  |  | |  |  |  |
| **Stage 3 trial - Harvesting** | **ZC3- Stage 3 trial - Harvesting** |  |  |  | |  |  |  |
| *ZC3 Labour cost of Stage 3 trial-Harvesting per m2* |  |  |  |  | |  |  |  |
| **Stage 3 trial - Seed processing** | **ZC4- Stage 3 trial - Seed processing** |  |  |  | |  |  |  |
| *ZC4 Cost of Cloth bag- Stage 3 trial-Harvesting & Seed processing per unit* |  |  |  |  | |  |  |  |
| *ZC4 Cost of Gunny bag- Stage 3 trial- Seed processing per unit* |  |  |  |  | |  |  |  |
| *ZC4 Labour cost of Stage 3 trial- Seed processing per m2* |  |  |  |  | |  |  |  |
| **Stage 3 trial - Post-harvest Observations** | **ZC5- Stage 3 trial - Post-harvest Observations** |  |  |  | |  |  |  |
| *ZC5 Cost of Seed Packet label- Stage 3 trial- Post-harvest Observations per unit* |  |  |  |  | |  |  |  |
| *ZC5 Cost of Paper envelop- Stage 3 trial- Post-harvest Observations per unit* |  |  |  |  | |  |  |  |
| *ZC5 Labour cost of Stage 3 trial - Post-harvest Observations per m2* |  |  |  |  | |  |  |  |
|  |  |  |  |  | |  |  |  |
| **Stage 4 trial** |  |  |  |  | |  |  |  |
| *Stage 4 trial- Cost of Fuel for monitoring trial- Site visit per trip* |  |  |  |  | |  |  |  |
| *Stage 4 trial- Cost of Fuel for monitoring trial- Site visit per trip* |  |  |  |  | |  |  |  |
| *Stage 4 trial- Cost of Fuel for monitoring trial- Site visit per trip* |  |  |  |  | |  |  |  |
| *Stage 4 trial- Cost of Land lease - Site per m2* |  |  |  |  | |  |  |  |
| *Stage 4 trial- Cost of Land lease - Site per m2* |  |  |  |  | |  |  |  |
| *Stage 4 trial- Cost of Land lease - Site per m2* |  |  |  |  | |  |  |  |
| *1 Breeder per diem per day-outstation* |  |  |  |  | |  |  |  |
| *1 Field technician per diem per day-outstation* |  |  |  |  | |  |  |  |
| *1 Driver per diem per day-outstation* |  |  |  |  | |  |  |  |
| *ZD1 Labour cost of Stage 4 trial- Observations at Flowering per m2* |  |  |  |  | |  |  |  |
| **Stage 4 trial- Observations at Pre-harvest** | **ZD2- Stage 4 trial- Observations at Pre-harvest** |  |  |  | |  |  |  |
| *ZD2 Labour cost of Stage 4 trial- Observations at Pre-harvest per m2* |  |  |  |  | |  |  |  |
| **Stage 4 trial- Harvesting** |  |  |  |  | |  |  |  |
| *ZD3 Labour cost of Stage 4 trial- Harvesting per m2* |  |  |  |  | |  |  |  |
| **Stage 4 trial- Seed processing** | **ZD4- Stage 4 trial- Seed processing** |  |  |  | |  |  |  |
| *ZD4 Cost of Cloth bag- Stage 4 trial-Harvesting & Seed processing per unit* |  |  |  |  | |  |  |  |
| *ZD4 Cost of Gunny bag- Stage 4 trial-Seed processing per unit* |  |  |  |  | |  |  |  |
| *ZD4 Labour cost of Stage 4 trial- Seed processing per m2* |  |  |  |  | |  |  |  |
| **Stage 4 trial- Post-harvest Observations** | **ZD5- Stage 4 trial- Post-harvest Observations** |  |  |  | |  |  |  |
| *ZD5 Cost of Seed Packet label- Stage 4 trial- Post-harvest Observations per unit* |  |  |  |  | |  |  |  |
| *ZD5 Cost of Paper envelop- Stage 4 trial- Post-harvest Observations per unit* |  |  |  |  | |  |  |  |
| *ZD5 Labour cost of Stage 4 trial- Post-harvest Observations per m2* |  |  |  |  | |  |  |  |
|  |  |  |  |  | |  |  |  |
| **Stage 5 trial** |  |  |  |  | |  |  |  |
| *Stage 5 trial- Cost of Fuel for monitoring trial- Site visit per trip* |  |  |  |  | |  |  |  |
| *Stage 5 trial- Cost of Fuel for monitoring trial- Site visit per trip* |  |  |  |  | |  |  |  |
| *Stage 5 trial- Cost of Fuel for monitoring trial- Site visit per trip* |  |  |  |  | |  |  |  |
| *Stage 5 trial- Cost of Land lease - Site per m2* |  |  |  |  | |  |  |  |
| *Stage 5 trial- Cost of Land lease - Site per m2* |  |  |  |  | |  |  |  |
| *Stage 5 trial- Cost of Land lease - Site per m2* |  |  |  |  | |  |  |  |
| *1 Breeder per diem per day-outstation* |  |  |  |  | |  |  |  |
| *1 Field technician per diem per day-outstation* |  |  |  |  | |  |  |  |
| *1 Driver per diem per day-outstation* |  |  |  |  | |  |  |  |
| *ZE1 Labour cost of Stage 5 trial- Observations at Flowering per m2* |  |  |  |  | |  |  |  |
| **Stage 5 trial- Observations at Pre-harvest** | **ZE2- Stage 5 trial- Observations at Pre-harvest** |  |  |  | |  |  |  |
| *ZE2 Labour cost of Stage 5 trial- Observations at Pre-harvest per m2* |  |  |  |  | |  |  |  |
| **Stage 5 trial- Harvesting** | **ZE3- Stage 5 trial- Harvesting** |  |  |  | |  |  |  |
| *ZE3 Cost of Gunny bag- Stage 5 trial- Harvesting per unit* |  |  |  |  | |  |  |  |
| *ZE3 Labour cost of Stage 5 trial -Harvesting per m2* |  |  |  |  | |  |  |  |
| **Stage 5 trial- Seed processing** | **ZE4- Stage 5 trial- Seed processing** |  |  |  | |  |  |  |
| *ZE4 Cost of Cloth bag- Stage 5 trial-Harvesting & Seed processing per unit* |  |  |  |  | |  |  |  |
| *ZE4 Cost of Gunny bag- Stage 5 trial Seed processing per unit* |  |  |  |  | |  |  |  |
| *ZE4 Labour cost of Stage 5 trial- Seed processing per m2* |  |  |  |  |  | |  |  |
| **Stage 5 trial- Post-harvest Observations** | **ZE5- Stage 5 trial- Post-harvest Observations** |  |  |  | |  |  |  |
| *ZE5 Cost of Seed Packet label- Stage 5 trial- Post-harvest Observations per unit* |  |  |  |  | |  |  |  |
| *ZE5 Cost of Paper envelop- Stage 5 trial- Post-harvest Observations per unit* |  |  |  |  | |  |  |  |
| *ZE5 Labour cost of Stage 5 trial- Post-harvest Observations per m2* |  |  |  |  | |  |  |  |
|  |  |  |  |  | |  |  |  |
| **On-Farm Trial** |  |  |  |  | |  |  |  |
| *Cost of On-Farm Trial Test per variety* |  |  |  |  | |  |  |  |
|  |  |  |  |  | |  |  |  |
